# 2-Bromopalmitate treatment attenuates senescence phenotype in human adult cells - possible role of palmitoylation

**DOI:** 10.18632/aging.206080

**Published:** 2024-08-23

**Authors:** Adam Krzystyniak, Agata Gluchowska, Agata Pytyś, Magdalena Dudkowska, Tomasz Wójtowicz, Alicja Targonska, Dorota Janiszewska, Ewa Sikora, Grazyna Mosieniak

**Affiliations:** 1 Laboratory of Molecular Bases of Aging, Nencki Institute of Experimental Biology, Warsaw, Poland; 2 Laboratory of Cell Biophysics, Nencki Institute of Experimental Biology, Warsaw, Poland; 3 Laboratory of Cytometry, Nencki Institute of Experimental Biology, Warsaw, Poland; 4 Laboratory of Calcium Binding Protein, Nencki Institute of Experimental Biology, Warsaw, Poland

**Keywords:** cell senescence, vascular smooth muscle cell, palmitoylation, 2-BP, DNA damage

## Abstract

Cells may undergo senescence in response to DNA damage, which is associated with cell cycle arrest, altered gene expression and altered cell morphology. Protein palmitoylation is one of the mechanisms by which the DNA damage response is regulated. Therefore, we hypothesized that protein palmitoylation played a role in regulation of the senescent phenotype. Here, we showed that treatment of senescent human vascular smooth muscle cells (VSMCs) with 2-bromopalmitate (2-BP), an inhibitor of protein acyltransferases, is associated with changes in different aspects of the senescent phenotype, including the resumption of cell proliferation, a decrease in DNA damage markers and the downregulation of senescence-associated β-galactosidase activity. The effects were dose dependent and associated with significantly decreased total protein palmitoylation level. We also showed that the senescence-modifying properties of 2-BP were at least partially mediated by the downregulation of elements of DNA damage-related molecular pathways, such as phosphorylated p53. Our data suggest that cell senescence may be regulated by palmitoylation, which provides a new perspective on the role of this posttranslational modification in age-related diseases.

## INTRODUCTION

Cellular senescence is a state of cell cycle arrest that occurs in response to various stressors, including DNA damage, and it involves functional and morphological alterations in the cell [1]. The double-strand DNA damage response (DDR) pathway, which controls the manifestation of the stress-induced senescence phenotype, canonically involves the activation of ataxia telangiectasia mutated kinase (ATM), which in turn phosphorylates histone H2AX and recruits proteins involved in DNA damage repair, such as 53BP1 [2]. ATM activation also leads to the phosphorylation and stabilization of p53, which in its active form translocates to the nucleus, where it initiates the expression of proteins involved in cell cycle arrest, such as p21 [3]. Furthermore, nuclear p53 activates autophagy by inhibiting mTOR [4]. As a consequence of activation of the above-mentioned signaling pathways, senescent cells display structural and functional changes, including changes in nuclear architecture, which are at least partially mediated by the loss of high-mobility group box 1 (HMGB1) and lamin B1 (LMNB1). HMGB1 is an abundant DNA binding protein that regulates chromatin structure. When DNA is damaged, HMGB1 is translocated to the cytoplasm and released extracellularly as a proinflammatory molecule [5]. LMNB1 is an integral part of the nuclear envelope, and its downregulated expression following p53 activation is a reliable marker of senescence [6]. However, the most widely used marker of senescence is the activity of senescence-associated β-galactosidase (SA-β-gal), which increases significantly with the buildup of β-galactosidase-rich lysosomal content in senescent cells [7].

Recently it has been shown that S-acylation, commonly referred to as S-palmitoylation or simply palmitoylation, may play a key role in the regulation of the DDR. Palmitoylation is a type of posttranslational modification of proteins mediated by protein acyltransferases (PATs) with zinc-finger and aspartate–histidine–histidine–cysteine (DHHC) motifs that catalyze the reversible addition of palmitate to specific cysteine (Cys) residues of a protein. Cao et al. [8] showed that treatment of primary mouse embryonic fibroblasts (MEFs) with the irreversible nonselective PAT inhibitor 2-bromopalmitate (2-BP) caused an aberrant response to DNA damage induced by doxorubicin. The global palmitoylation inhibition induced by 2-BP treatment abrogated cell cycle arrest and caused increased formation of DNA damage foci containing γH2AX and 53BP1. These phenotypic changes were mediated by defective ATM and p53 activation. The authors also found that ablation of zinc finger DHHC-type palmitoyltransferase 16 (ZDHHC16) produced similar results and revealed the link between palmitoylation and the DNA damage response [8]. Another elegant recent study showed that ZDHHC1 mediated the nuclear translocation of p53. Palmitoylation was necessary for p53 nuclear trafficking and subsequent pathway activation [3].

The important role of palmitoylation in the DNA damage response led us to speculate about the role of palmitoylation in the regulation of cellular senescence. Thus, we decided to investigate how the global inhibition of PATs with 2-BP affects different aspects of cellular senescence. To accomplish this, we used a model of DNA damage-induced cellular senescence that we have extensively studied [9]. In this model, adult human vascular smooth muscle cells (VSMCs) are treated with doxorubicin. By analyzing phenotypic changes at the structural, functional and molecular levels over time, we showed that the inhibition of palmitoylation may participate in the regulation of the senescence phenotype. We found that treatment with 50 μM 2-BP before, but not after, the induction of doxorubicin-mediated DNA damage modified some aspects of the senescent phenotype. This effect was associated with decreased levels of phosphorylated p53. To our knowledge, this study is the first report to show a possible role played by palmitoylation in the regulation of cell senescence.

## RESULTS

### 2-BP slows proliferation without inducing markers of senescence

Since protein palmitoylation has been shown to play a role in the regulation of the DNA damage response [8], we sought to determine whether the potent palmitoylation inhibitor 2-BP could affect the senescence phenotype of human VSMCs. We first determined the influence of 2-BP on non-senescent VSMCs. To analyze the effects of palmitoylation inhibition in our model, we treated proliferating VSMCs with increasing doses of 2-BP and analyzed the protein level of palmitoylation, cellular proliferation, DNA synthesis, DNA damage and SA-β-gal activity.

**Figure 1:**
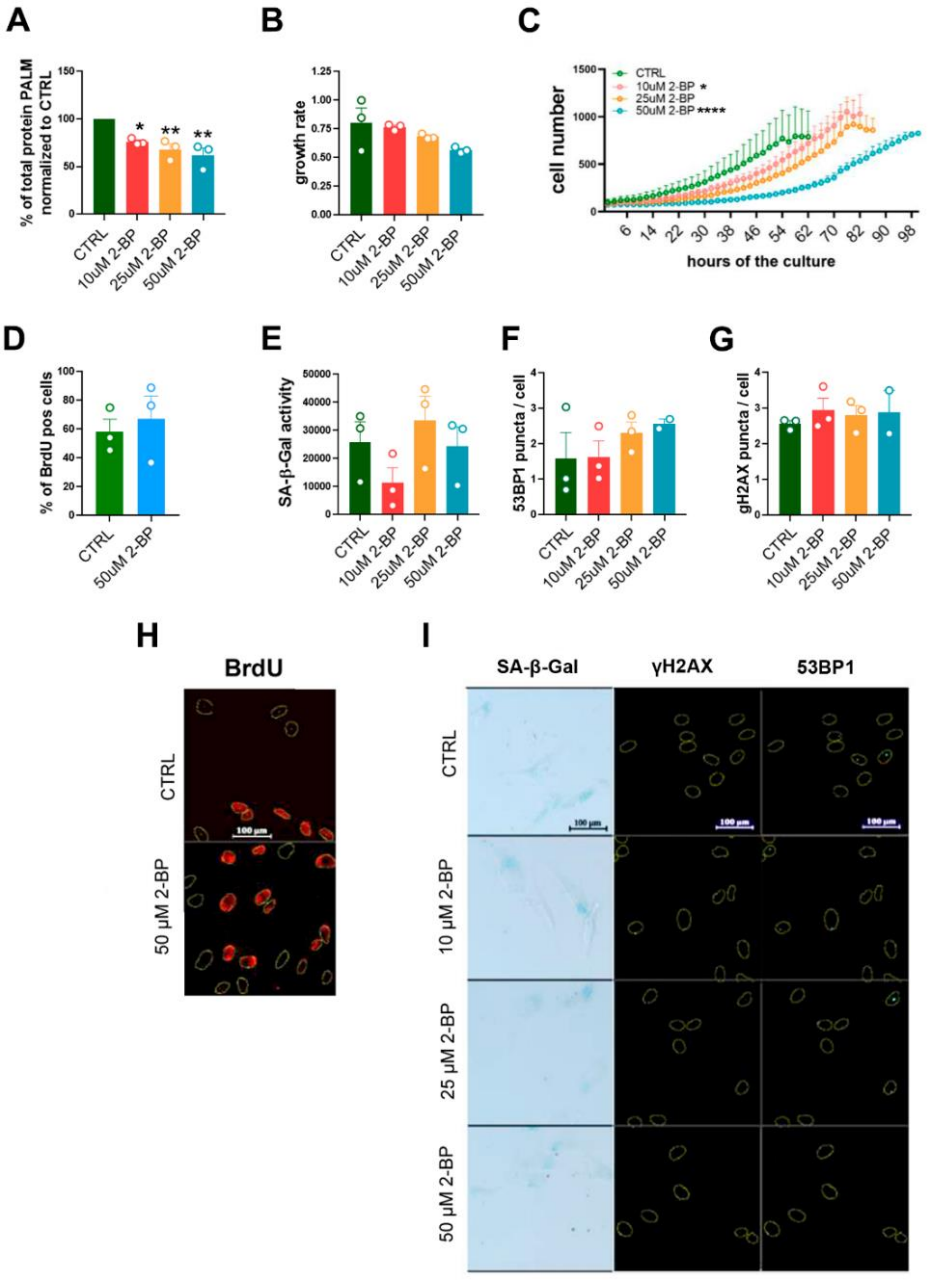
**The influence of 2-BP treatment on cell proliferation and senescence.** The graph shows the total protein palmitoylation normalized to the control after 24 h of 2-BP treatment (**A**). Analysis of the effects of different doses of 2-BP on the proliferation rate, as measured by growth rate calculations (**B**) and recording of cell numbers during a 4-day culture (**C**). Changes in the number of BrdU-positive cells (**D**), the level of SA-β-gal activity (**E**), and the number of DNA damage-associated 53BP1 foci (**F**) and γH2AX foci (**G**). Representative images of the measurements are shown in Panels (**H**) (BrdU incorporation) and (**I**) (SA-β-gal activity, 53BP1, γH2AX). The results are presented as the means ± SEMs from N=3 biological replicates. Statistical analysis was performed using one-way ANOVA or two-way ANOVA for (**C**), *p< 0.05, **p<0.01, ****p<0.0001.

As expected, 2-BP treatment was associated with a dose-dependent decrease in the total protein palmitoylation level in whole-cell lysates compared to the control, as detected by an acyl-biotinyl exchange assay (ABE) followed by Western blotting (Figure 1A and Supplementary Figure 1).

VSMCs are relatively fast proliferating cells that exhibit a gradually slowing of their proliferation rate within a few days of culture, which may result in the acquisition of some features of a senescent phenotype, such as the nonspecific activation of SA-β-gal. Therefore, we compared the results obtained in treated cells to those in untreated cells (CTRL) 48 h after seeding, when the cells retained a high proliferation rate.

Treatment of VSMCs with 2-BP decreased the proliferation rate of the cells (Figure 1B, 1C). However, we did not observe any changes in the number of BrdU-incorporating cells (Figure 1D, 1H), the expression of senescence markers, such as SA-β-gal activity (Figure 1E, 1I) or the number of 53BP1 or γH2AX foci, (Figure 1F, 1G, 1I), in 2-BP-treated cells. These results suggest that palmitoylation inhibition in growing cells does not induce permanent growth arrest.

### 2-BP treatment affects the development of a senescent phenotype in VSMCs treated with doxorubicin

**Figure 2:**
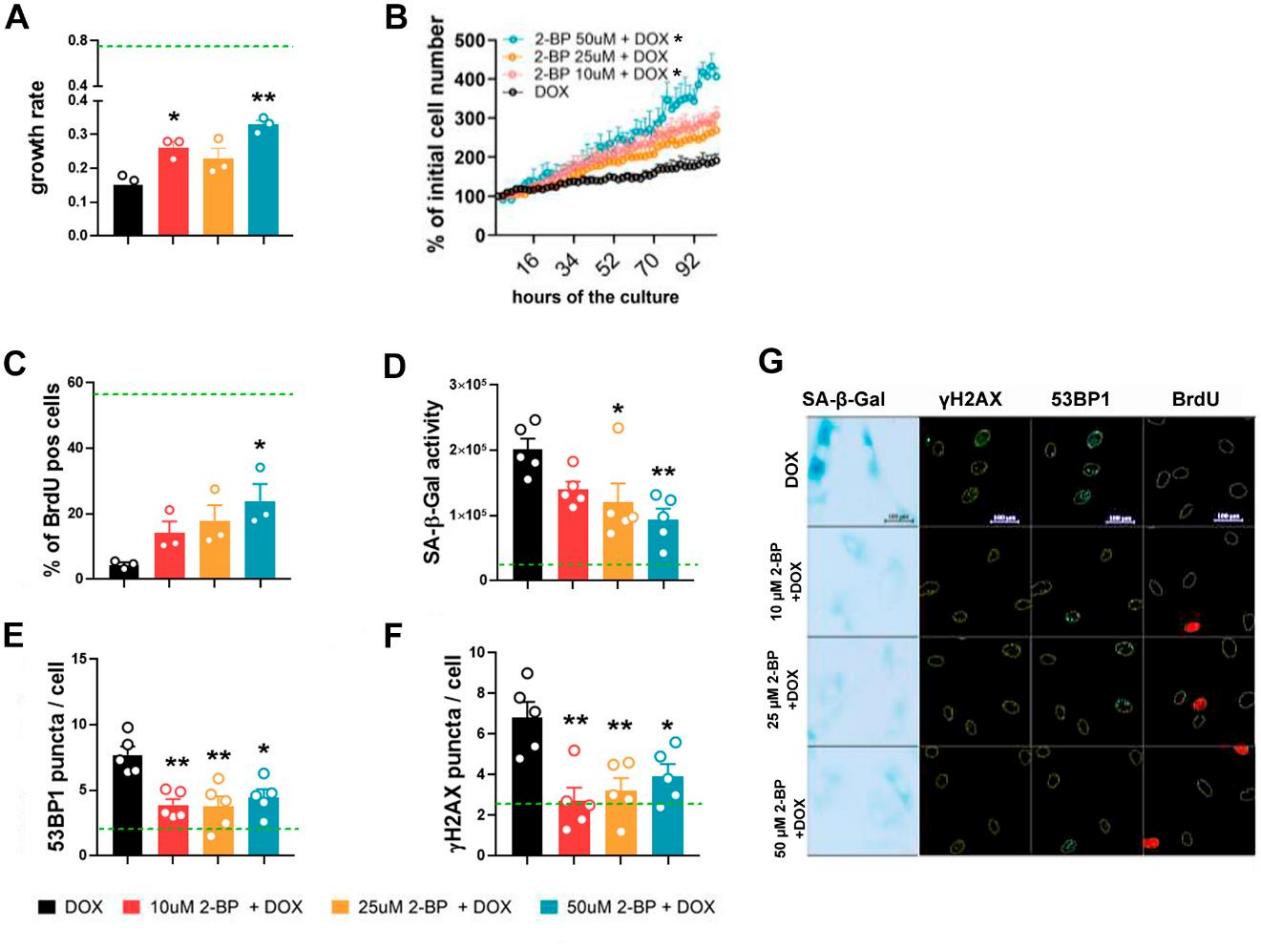
**2-BP treatment decreases the expression of senescence markers in doxorubicin-treated VSMCs.** Cells were pretreated with increasing doses of 2-BP for 24 h (pretreatment), washed and induced to senescence by a 2 h treatment with doxorubicin (DOX). The proliferation rate during a 4-day culture measured every 2 h was used to calculate the growth rate (**A**, **B**). Changes in the number of BrdU-positive cells (**C**), the level of SA-β-gal activity (**D**), and the number of DNA damage-associated 53BP1 (**E**) and γH2AX foci (**F**) 3 days after doxorubicin treatment in response to increasing doses of 2-BP. Green line marks the mean level of analyzed parameter in control, untreated cells. The results are shown as the means with SEM from N=3 or 5 biological replicates. Statistical analysis was performed using one-way ANOVA (comparison between DOX and 2-BP + DOX) or two-way ANOVA (for B), p≤ 0.05, **p≤0.01. Representative images of SA-β-gal activity and 53BP1 and γH2AX levels are shown in Panel (**G**).

The inhibition of protein palmitoylation with 2-BP has been shown to affect DNA damage-induced focus assembly/disassembly and to override cell cycle arrest in primary mouse embryonic fibroblasts [8]. Therefore, we hypothesized that 2-BP could influence the phenotypic transition from proliferating to senescent cells upon DNA damage caused by doxorubicin treatment. To prevent cytotoxicity in cultures co-treated with doxorubicin and 2-BP [8], we pretreated VSMCs with 2-BP for 24 h. The 2-BP was removed by washing, and fresh medium containing 1 μM doxorubicin (DOX) was added and left for 2 h to induce senescence according to our previously established protocol [9]. Three days after the initial 2-BP treatment, the levels of senescence markers were analyzed. We found that 2-BP partially released the inhibition of proliferation caused by doxorubicin (Figure 2A, 2B). Notably, the observed effect was dose dependent but not dose proportional. At least 24 palmitoyltransferases might be differentially affected by 2-BP treatment, which may result in an activity configuration-promoting response that is not necessarily proportional to the dose.

The changes in proliferation were reflected by an increase in the number of BrdU-positive cells compared to doxorubicin-treated cells (Figure 2C). Treatment with 2-BP was also associated with decreased activity of SA-β-gal in DOX-treated cells (Figure 2D). The expression of DNA damage markers, such as 53BP1 and γH2AX, quantified by the number of foci per nucleus (Figure 2E, 2F), decreased significantly in 2-BP-treated cells compared to the number in cells treated with doxorubicin alone, regardless of the concentration of the inhibitor. Representative images of SA-β-gal activity analysis and 53BP1, γH2AX, and BrdU immunostaining are shown in Figure 2G. We observed similar trends in the changes in the proliferation rate of doxorubicin-treated human fibroblasts upon 2-BP treatment, but these changes were not significant, which suggests that our findings are cell/dose specific (Supplementary Figure 2).

### The senescence-modifying activity of 2-BP is at least partially mediated by disruption of the DNA damage response

**Figure 3:**
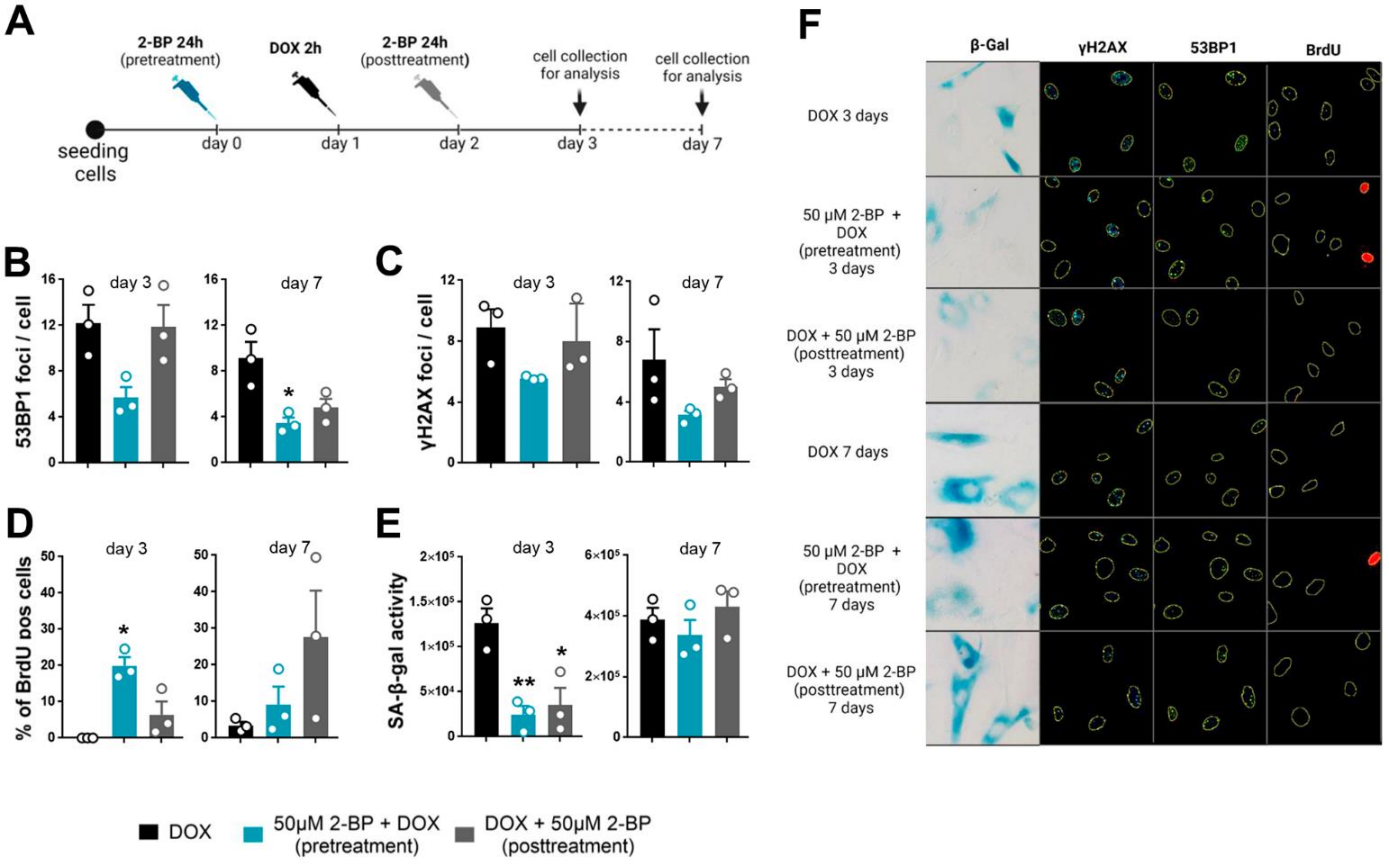
**The senescence-modifying effect of 2-BP is detected only if cells are treated before senescence induction.** VSMCs were treated with 2-BP for 24 h before (pretreatment) or after (posttreatment) doxorubicin (DOX)-mediated DNA damage induction. DNA damage was analyzed at different time points (3 and 7 days) following incubation with 2-BP. (**A**) Schematic representation of the experimental design. The graph shows the number of DNA damage-associated nuclear 53BP1 and γH2AX foci after doxorubicin treatment (**B**, **C**), the percentage of cells that incorporated BrdU (**D**), and SA-β-gal activity (**E**). The results are presented as the means ± SEMs from two separate experiments, each with N=3 biological replicates. Statistical analysis was performed using one-way ANOVA; *p< 0.05, **p<0.01. Representative images of SA-β-gal activity and 53BP1 and γH2AX levels are shown in Panel (**F**).

**Figure 4:**
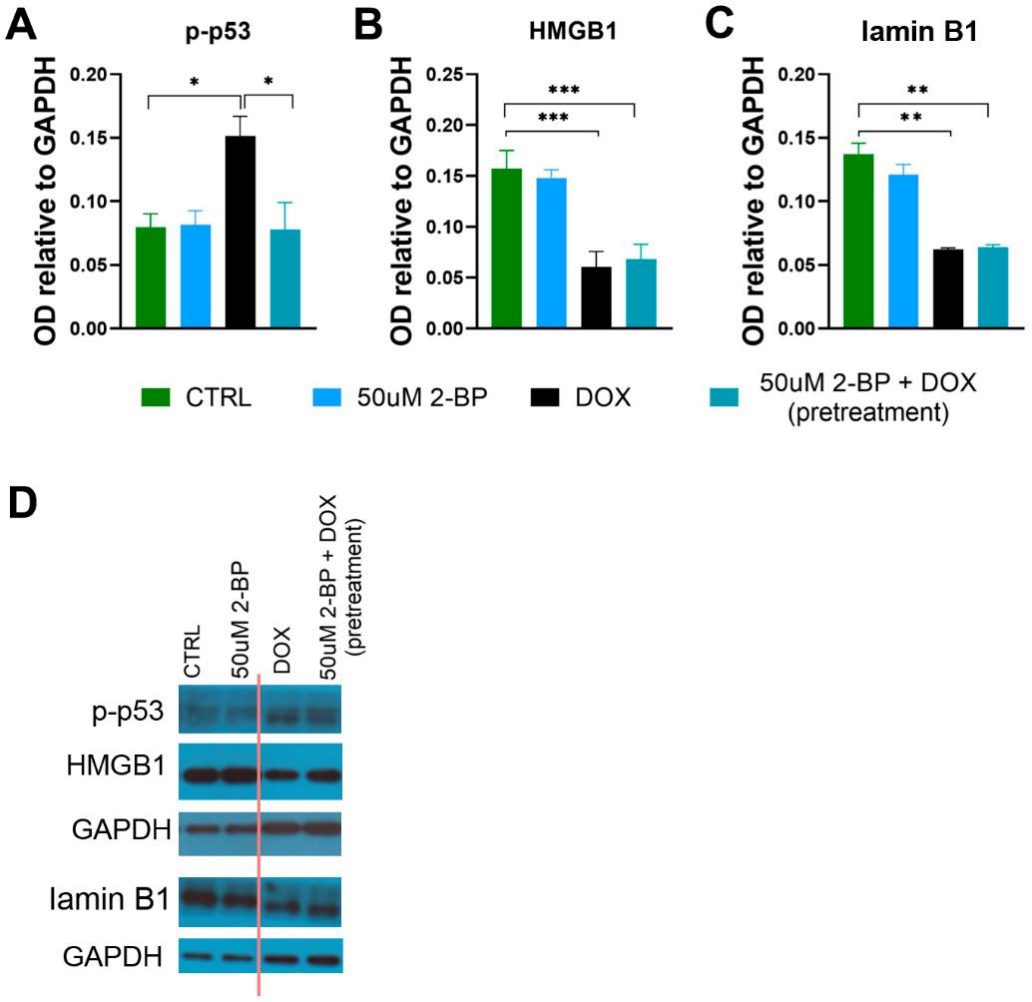
**The influence of 2-BP treatment on the levels of senescence markers.** The levels of p-p53 (**A**), HMGB1 (**B**), and lamin B1 (**C**) in cell extracts from VSMCs treated for 24 h with 2-BP before doxorubicin (DOX) treatment (pretreatment) or treated with 2-BP alone for the indicated times were analyzed via Western blotting. Representative Western blots (the cutting line of the blot is shown in red) (**D**). The results are presented as the means ± SEMs from N=3 biological replicates. Statistical analysis was performed using one-way ANOVA; *p< 0.05, **p<0.01, ***p<0.001.

It has been previously shown that 2-BP may affect the DNA damage response by blocking p53 activation and translocation to the nucleus [3, 9]. To determine whether the inhibition of senescence phenotype development by 2-BP is dependent on this mechanism, we treated cells with 50 μM 2-BP for 24 h before (pretreatment) or after (posttreatment) the induction of DNA damage with doxorubicin (Figure 3A). We hypothesized that cultured cells treated with 2-BP after the induction of DNA damage would not respond to phenotypic changes in the same way as the pretreatment variants because the DNA damage response pathway would already be initiated and p53 would be activated. We did not observe significant changes in the levels of DNA damage markers, such as the number of γH2AX and 53BP1 foci in the nuclei, or in the percentage of BrdU-positive cells in the posttreatment variant compared to doxorubicin-treated cells on day 3 or day 7. Cells pretreated with 2-BP before DOX treatment showed significantly fewer 53BP1 foci (day 7) and more BrdU-positive cells (day 3) compared to DOX-treated cells (Figure 3B–3D, 3F). Notably, the level of activity of SA-β-gal decreased significantly in pretreated and posttreated cells on day 3 regardless of differences in the levels of DNA damage markers and BrdU incorporation in pre- and posttreated variants. However, the initial decrease in SA-β-gal activity was no longer observed on day 7 (Figure 3E, 3F). Western blot (WB) analysis revealed a significant decrease in the p-p53 protein level caused by 2-BP pretreatment of DOX-treated cells (Figure 4A, 4D), but culturing the cells with 2-BP after DOX treatment (posttreatment variant) did not affect p-53 phosphorylation (Supplementary Figure 3).

As expected, we observed a significant decrease in HMGB1 and lamin B1 levels upon induction of senescence by doxorubicin treatment. However, the levels of these proteins remained unchanged upon treatment with 2-BP and doxorubicin (Figure 4B–4D). Similarly, 2-BP alone did not influence HMGB1 or lamin B1 levels in posttreated cells (Supplementary Figure 3).

Of note, doxorubicin treatment alone was associated with a decrease in the total protein palmitoylation level after 3 days. Notably, 2-BP and doxorubicin did not act synergistically in this regard (Supplementary Figure 4).

## DISCUSSION

Here we show for the first time that the global inhibition of the protein palmitoylation with 2-BP is associated with attenuation of the DNA damage-induced senescence phenotype of human VSMCs. Cells treated with 2-BP for 24 h before the induction of senescence had significantly decreased SA-β-gal activity; lower levels of DNA damage markers, such as γH2AX and 53BP1 foci and a partial reversal of cell cycle arrest. These changes were accompanied by decreased levels of phosphorylated p53, which is consistent with previously published results [9]. The lack of significant differences in the proliferation of cells treated with 2-BP after doxorubicin-induced senescence confirmed previously published results suggesting that palmitoylation is critical for the translocation of p53 to the nucleus [3]. Once p53 is translocated, it can no longer be affected by palmitoylation inhibition, which may explain the lack of significant changes in the levels of phosphorylated p53 in the posttreatment variant of our study (Supplementary Figure 3A). Notably, SA-β-gal activity significantly decreased in a dose-dependent manner when 2-BP was applied both before and after doxorubicin induction. This finding suggests that SA-β- gal activity may be regulated independently of the ATM-p53 axis. Indeed, SA-β-gal is a lysosomal-β-galactosidase protein that exhibits increased activity during senescence, although it is not necessary for the development of the senescence phenotype [7]. However, the mechanism by which palmitoylation affects the activity of this enzyme is not clear. 2-BP treatment has been shown to disturb the vacuolar ATPase-dependent lysosomal acidification pump in neurons via inhibition of the palmitoylation of one of its subunits [10]. Since acidification of intracellular organelles is critical for the regulation of autophagy [11], SA-β-gal activity could be associated with changes in autophagy as a result of 2-BP treatment.

In senescent cells pretreated with 2-BP, we observed a decrease in the number of γH2AX and 53BP1 foci. The lack of dose dependence of this effect suggests that it is sensitive to a very low level of palmitoylation inhibition. Our observations seem to contrast with those described in the report by Cao et al., in which 2-BP treatment was shown to increase the doxorubicin-induced number of γH2AX and 53BP1 foci [8]. However, due to multiple differences in the experimental setup, such as cell types (human VSMCs vs. MEFs), observation time windows (3-7 days vs. 0-24 h), duration of exposure to doxorubicin (2 h vs. 24 h) and, most importantly, coincubation of doxorubicin with 2-BP (which we also found to be highly detrimental to cells even at a 50 μM concentration; results not shown), our data are not necessarily contradictory.

Cell senescence is a dynamic process, and our study revealed the transient nature of the senescence phenotype-modifying properties of a palmitoylation inhibitor. Almost all parameters that reached statistical significance compared to the doxorubicin-treated control on day 3 of the experiment were no longer different on day 7. The transient nature of the effect may be the result of not only the exhaustion of 2-BP inhibitory activity but also the naturally occurring recovery from DNA damage in cells after treatment with doxorubicin alone, which was used as a reference.

Regardless of the treatment regimen, 2-BP did not influence the expression of other markers of senescence, such as HMGB1 or lamin B1. Both of these proteins are directly engaged in sustaining the functional and structural integrity of the nucleus, which is compromised by doxorubicin treatment. However, the decrease in lamin B1 expression in senescent cells has been shown to be mediated by p53 activation [6], which is downregulated by 2-BP. Perhaps the remaining activity of p53 was sufficient to induce a decrease in lamin B1 expression and a phenotypic shift.

Our data provide important insight into the possible mechanisms underlying the recently reported senescence-inducing effects of palmitate. Experiments using several *in vitro* models involving adipocytes [12], cardiac fibroblasts [13] and neurons [14] have shown that the treatment of cells with palmitate triggers a senescent phenotype. Palmitic acid is sourced partially from dietary intake [15]. Insulin also affects the palmitoylation of hundreds of proteins in human endothelial cells [16] and brain cells, which ultimately affects brain plasticity and memory [17]. Therefore, diet/hunger may have an important impact on the availability of palmitate and the extent to which proteins are regulated by the covalent attachment of this fatty acid. However, this process is poorly understood. Excess palmitate in the brain has negative consequences for cognition and may result in inflammation, astroglial and microglial activation, and tumor necrosis factor α (TNF-alpha) signaling [18]. In addition, a palm oil-rich diet was shown to affect the murine liver proteome and S-palmitoylome, leading to palmitate accumulation [19]. Therefore, in the future, it would be interesting to study the effect of the interaction between palmitoylation and diet/hunger on lifespan in high-fat diet and caloric restriction models.

Given the pioneering nature of our study, some limitations and multiple questions are unavoidable. First and foremost, we used 2-BP, which is a broad-spectrum inhibitor of PATs. Although the posttreatment variant in our study provides some evidence that the senescence-modifying action of 2-BP is specific to certain molecular pathways, we do not know which PATs are involved in this process. Our data also revealed that 2-BP treatment resulted in a significant and dose-dependent decrease in the total protein palmitoylation level compared to the control group. However, doxorubicin itself also reduced the total protein palmitoylation level after 3 days to a level comparable to that achieved by treatment with 50 μM 2-BP. Notably, 2-BP and doxorubicin do not act synergistically in this regard. These seemingly conflicting data may be explained by the fact that cells in different biological contexts may regulate protein function by downregulating and upregulating palmitoylation. The ABE method we employed in our study provides information about the average palmitoylation level of all proteins. However, as shown previously, the average palmitoylation level may not differ between treatment groups, but the palmitoylation levels of individual proteins may differ significantly [20]. Analysis of the palmitoylation levels of individual proteins in senescent cells and how they change upon senescence-modifying treatments could reveal possible molecular targets for the treatment of aging-associated diseases.

## CONCLUSIONS

For the first time, the present study revealed a critical role for protein palmitoylation in the development of a DNA damage-induced senescence phenotype. Aging and age-related disorders affect a growing population of people worldwide and are among the major challenges of modern science and medicine [21]. Recently, senescent cells have become a very promising target for organism rejuvenation [22, 23]. The results presented here advance our understanding of the regulation of senescence and may contribute to the discovery of new molecular targets for senotherapy.

## MATERIALS AND METHODS

### Cell culture

Human vascular smooth muscle cells (VSMCs) or human fibroblasts were purchased from ATCC (Manassas, VA, USA) (normal diploid cells derived from young males, at least from three different donors). VSMC were cultured in vascular cell basal medium (ATCC) supplemented as defined by the manufacturer and kept in a humidified atmosphere (37° C and 5% CO_2_ in the air). Cells were seeded at a density of 3000/cm^2^ and passaged every 3-4 days. To induce senescence, 24 h after seeding, the cells were treated with 1 μM doxorubicin for 2 h. After doxorubicin treatment, the cells were washed with warm PBS and cultured in fresh medium for 3 or 7 days. For experiments with 2-bromopalmitate, cells were treated for 24 h with 10, 25 or 50 μM 2-BP. 2-BP stock solutions, at concentrations of 10, 25 and 50 mM, were prepared in EtOH immediately before use and diluted to working solutions in the culture medium. Depending on the experimental variant, the cells were incubated with 2-BP before (pretreatment) or after (posttreatment) doxorubicin treatment. The 2-BP-treated cells were washed with warm PBS and cultured in fresh culture medium.

### Monitoring of proliferation

We monitored the proliferation of unstained VSMCs or fibroblasts in different experimental groups using the IncuCyte SX1 Live-Cell Analysis System (Sartorius, Göttingen, Germany). The system allows bright field and fluorescence imaging of live cells in culture plates inside a standard incubator (37° C, 5% CO_2_ and 100% humidity). The system is equipped with algorithms allowing the stain-free analysis of cell proliferation by automatic cell counting. BF images of a 96-well plate were collected every 2 h. Integrated IncuCyte software was used to analyze the results.

### SA-β-gal staining and analysis

The detection of SA-β-gal activity was performed according to Dimri et al. [24]. Briefly, cells were fixed with 2% formaldehyde and 0.2% glutaraldehyde in PBS, washed, and exposed overnight at 37° C to a solution containing 1 mg/ml 5-bromo-4-chloro-3-indolyl-b-D-galactopyranoside, 5 mM potassium ferrocyanide, 5 mM potassium ferricyanide, 150 mM NaCl, 2 mM MgCl2, and 0.02 M phosphate buffer, pH 6.0. All of the agents used were purchased from Sigma Aldrich (St. Louis, MO, USA). For imaging, the cells were embedded in 4 μl of mounting medium supplemented with DAPI (Abcam, Cambridge, UK) and mounted on glass slides. Images were taken at least 10 min after embedding the cells in the medium with transmitted light or fluorescence (excitation 340-380 nm, emission 435-485 nm), using a Nikon Eclipse Ti-U fluorescence microscope and Nikon Digital Sight DS-U3 camera (Nikon, Tokyo, Japan) with a 20x objective at a resolution of 2560 x 1920 pixels and exposure set at 15 ms (BF images) with 30% of the maximum lamp intensity. For SA-β-gal staining, we analyzed at least 100 cells from 3-5 independent biological replicates using a previously described automatic method [25]. Briefly, BF (SA-β-gal) and fluorescent (DAPI) images were analyzed using a Fiji-based platform developed and validated by us, which automatically selects areas of the image with blue, green, and red color intensities above the experimentally determined color threshold (Hue = 117-185; Saturation = 80-255; Brightness = 0-255). The area and intensity of the SA-β-gal signal were used to calculate the integrated density.

### BrdU incorporation and staining

The cells were treated with 10 μM BrdU for at least 18 h of culture then washed with PBS and fixed in ice-cold 70% ethanol. Fixed cells were washed with 0.5% Triton X-100 (Sigma-Aldrich, St. Louis, MO, USA) in PBS, incubated in 2 N HCl for 30 min, washed twice with PBS, incubated for 1 min in 0.1 M borax solution (Sigma-Aldrich), washed twice in PBS again and incubated with primary anti-BrdU antibody (Becton Dickinson, Franklin Lakes, NJ, USA) diluted 1:120 in 1% BSA 0.5% Tween-20 in PBS for 1 h. After incubation, the cells were washed twice with 0.5% Tween-20 in PBS and incubated with a secondary antibody conjugated to a fluorochrome (Thermo Fisher Scientific, Waltham, MA, USA). The stained cells were mounted on a microscope slide with mounting medium supplemented with DAPI (Abcam) and imaged under an Eclipse fluorescence microscope (Nikon, Tokyo, Japan). The number of BrdU-positive cells and the total cell number (based on DAPI staining) were calculated using ImageJ (National Institutes of Health, Bethesda, MD, USA).

### Immunostaining of γH2AX and 53BP1

The cells were washed with PBS, fixed in 4% paraformaldehyde, permeabilized in 0.3% Triton X-100 in PBS then blocked in PBS supplemented with 2% BSA, 1.5% normal goat serum and 0.1% Triton X-100 for 1 hour. Permeabilized cells were incubated with the following primary antibodies: anti-53BP1 (NB100-304, Novus, USA), anti-γH2AX (ab26350, Abcam) diluted in blocking solution for 2 hours. The sections were then washed with PBS supplemented with 0.1% Triton X-100 and incubated with Alexa Fluor 488-conjugated anti-rabbit, Alexa Fluor 555-conjugated anti-mouse or Alexa Fluor 594-conjugated anti-guinea pig secondary antibody in blocking solution for 1 h. The cells were mounted on a microscope slide with mounting medium supplemented with DAPI (Abcam). Images of cells stained for γH2AX and 53BP1 were obtained with an Eclipse fluorescence microscope (Nikon, Tokyo, Japan). Images of cells stained for p62 were obtained using an Sp8 confocal (Leica, Wetzlar, Germany) microscope with an HPPL APO CS2 63x/1.4° oil objective, a laser-405 diode (Pico Quant, Berlin, Germany) and white light (NKT Photonics, Birkerød, Denmark). The interval of the optical section was 0.5 μm, and the pixel section was 0.180 μm. The γH2AX and 53BP1 foci were analyzed in each cell nucleus (based on DAPI staining). Analysis was performed using ImageJ (National Institutes of Health, Bethesda, MD, USA).

### Acyl-biotinyl exchange assay

To analyze changes in protein palmitoylation levels, an acyl-biotinyl exchange (ABE) assay [26] was used. Cells were lysed and homogenized using a Dounce homogenizer in buffer containing 50 mM Tris HCl (pH 7.5), 150 mM NaCl, 1 mM EDTA, 4% SDS and 1% Triton X-100. Proteins were reduced with 10 mM TCEP (tris(2-carboxyethyl)phosphine), and the samples were incubated for 16 h at 4° C with 50 mM N-ethylmaleimide (NEM) to block free thiol groups. Proteins were precipitated using ice-cold ethanol to remove unreacted NEM. The pellets were resuspended in the same buffer, and the samples were divided in half. Both halves were treated with 400 μM thiol-reactive biotinylation reagent HPDP-biotin (N-[6-(biotinamido)hexyl]-3’-(2’-pyridyldithio)propionamide). One half was treated with 1 M hydroxylamine to cleave thioester-linked palmitoyl moieties and expose newly formed thiols to HPDP-biotin. The other half was treated with Tris buffer (pH 7.5) as a control to nonspecific binding of HPDP-biotin. Biotinylated proteins were analyzed with Western blotting.

### Western blotting

Cultured cells were lysed in reducing sample buffer containing 125 mM Tris–HCl (pH 6.8), 4% SDS, 20% glycerol, 100 mM DTT, and 0.2% bromophenol blue and denatured for 10 min at 95° C. The total protein concentration was estimated using a bicinchoninic acid (BCA) protein assay kit, and 20 μg of each sample was loaded on a gel. Protein lysates were separated by SDS-PAGE using 4-12% (w/v) Bis-Tris gels. The separated proteins were transferred to nitrocellulose membranes (Amersham GE Healthcare, UK) and blocked in 5% non-fat powdered milk in TBS containing 0.1% Tween-20 (TBST) for 1 h. The membranes were incubated overnight with primary antibodies against p-p53, p-ATM, HMGB1, LMB-1 and GAPDH, washed in TBST, incubated with horseradish peroxidase (HRP)-conjugated secondary antibodies (anti-mouse or anti-rabbit, Dako Denmark A/S) at a 1:2000 dilution in 5% milk, washed and visualized with enhanced chemiluminescence (ECL) (Thermo Fisher Scientific). Signals were detected using X-ray film or ChemiDoc (Bio-Rad, Hercules, CA, USA) and analyzed with ImageJ (National Institutes of Health, Bethesda, MD, USA).

### Statistical analysis

Statistical significance was analyzed using one-way ANOVA or two-way ANOVA, as indicated in the figure legends. All statistical tests were performed using GraphPad Prism 9 (GraphPad, San Diego, CA, USA). The data are presented as the means +/− SEMs.

## Supplementary Material

Supplementary Figures
