# Supplementary material for: 2-Bromopalmitate treatment attenuates senescence phenotype in human adult cells - possible role of palmitoylation
Source: Aging (Albany NY). 2024 Aug 23;16(16):11796–808. doi: 10.18632/aging.206080 (PMC11386925; doi:10.18632/aging.206080)
Supplement: Supplementary Figures [file aging-16-206080-s001.pdf]

SUPPLEMENTARY FIGURES

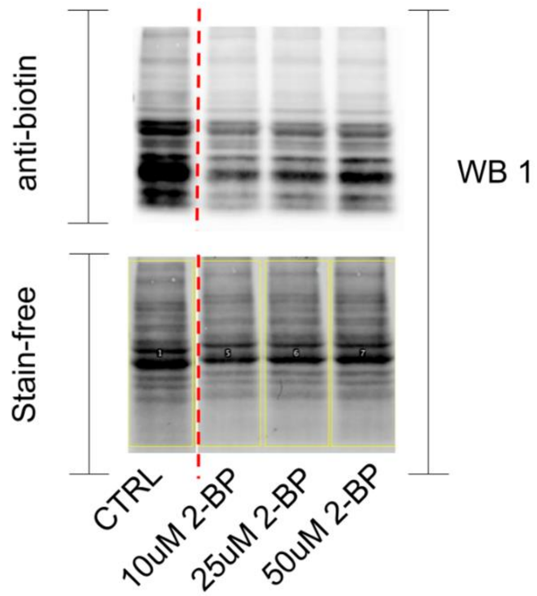

Supplementary Figure 1. Representative Western blot images of cell lysates from non-senescent human VSMCs analyzed 24h after treatment with 2-BP at different doses. Inhibition of total palmitoylation level in VSMCs treated with 2-BP. Palmitoylation levels were analyzed using acyl-biotinyl exchange assay (ABE).

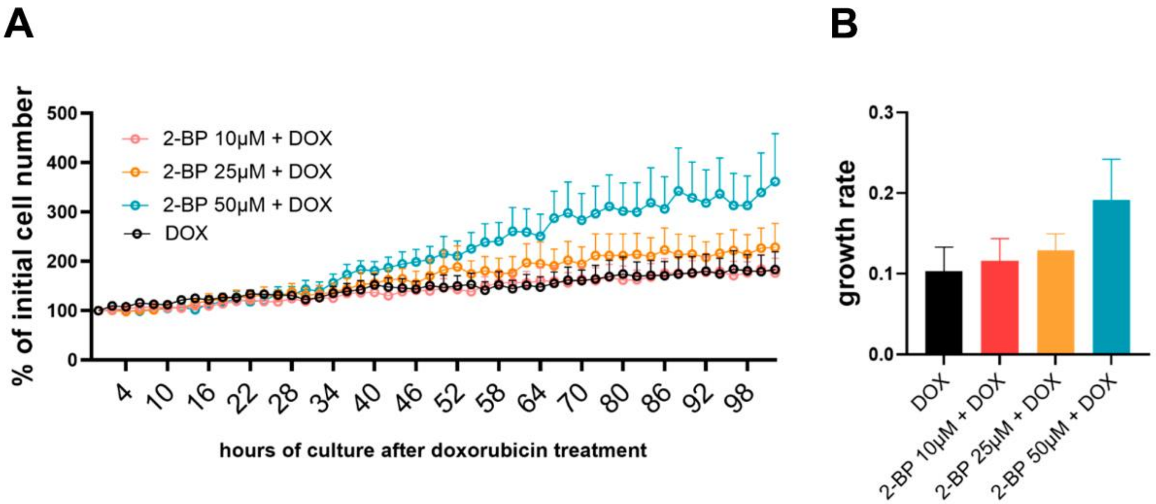

Supplementary Figure 2. Normal human fibroblasts treated with 2-BP for 24h before doxorubicin (DOX) treatment. Proliferation rates during a 4-day culture were measured every 2h (**A**) and growth rate calculated based on proliferation data (**B**). Results are shown as mean with SEM from N=3 technical replicates. Data were analyzed using one-way ANOVA, no significant differences were revealed.

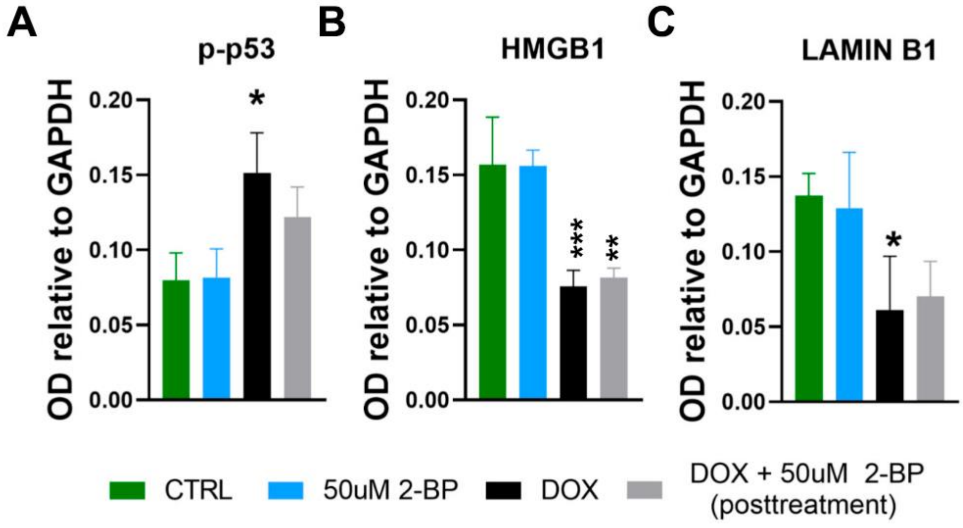

Supplementary Figure 3. The level of proteins associated with senescence phenotype. p-p53 (**A**), HMGB1 (**B**), lamin B1 (**C**) in VSMCs treated with 2-BP for 24h after (posttreatment) doxorubicin (DOX) mediated induction of DNA damage, analyzed 3 days after incubation with 2-BP. Results are shown as mean with SEM from N=3 biological replicates. Statistical analysis performed using one-way ANOVA, * significantly different from control cells cultured for 48 after seeding. (CTRL) (*p<0.05, **p<0.01, ***p<0.001).

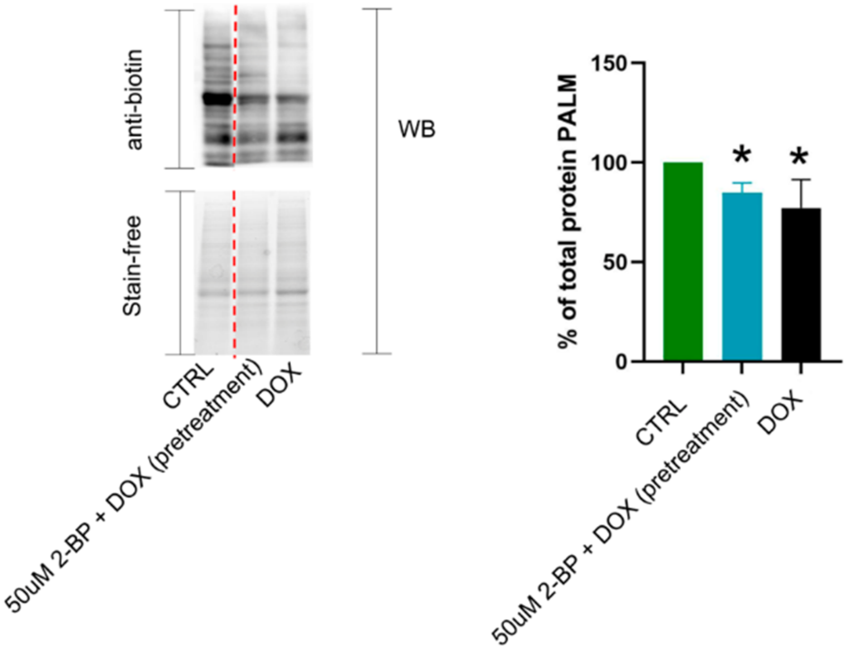

Supplementary Figure 4. Representative Western blot images of cell lysates from human VSMCs treated with 50 μM of 2-BP for 24h and then induced to senesce by treatment with 1 μM doxorubicin for 2h or treated with 1 μM doxorubicin for 2h alone (DOX) and harvested 48h after DOX treatment. Untreated control cells cultured for 48h (CTRL) were used as reference. Stain-free total protein visualization was used as a loading control. Lanes with samples that were not used for analysis were cut out from the membrane (the cutting line is shown in red). Graph presents quantification of the total protein palmitoylation level. Results are mean with SEM from N=3 biological replicates. Statistical analysis performed using Student's t-test, * significantly different from CTRL (*p< 0.05).
